# P-1362. In vivo Efficacy of an Aztreonam-Avibactam Human Simulated Regimen against Stenotrophomonas maltophilia in the Murine Thigh Infection Model: Decision Support for Susceptibility Breakpoint Determination

**DOI:** 10.1093/ofid/ofaf695.1549

**Published:** 2026-01-11

**Authors:** Yakun Fu, Aliaa Fouad, David P Nicolau, Joseph L Kuti

**Affiliations:** Hartford hospital, Hartford, CT; Hartford Hospital, Farmington, CT; Hartford Hospital, Farmington, CT; Hartford Hospital, Farmington, CT

## Abstract

**Background:**

There are few antibiotic treatments available for severe infections caused by *Stenotrophomonas maltophilia* (STM). Aztreonam-Avibactam (ATM-AVI) is a beta-lactam/beta-lactamase inhibitor antibiotic recently approved by the FDA for complicated intra-abdominal infections caused by Enterobacterales. ATM-AVI also has *in vitro* activity against STM with an MIC_90_ of 4/4 mg/L (MIC range: 0.5/4 to > 16/4 mg/L). Understanding at which MIC the approved dosage of ATM-AVI is likely to be effective can assist setting susceptibility breakpoints and ultimately provide guidance to test this drug against STM in the clinic.

**Figure 1:**
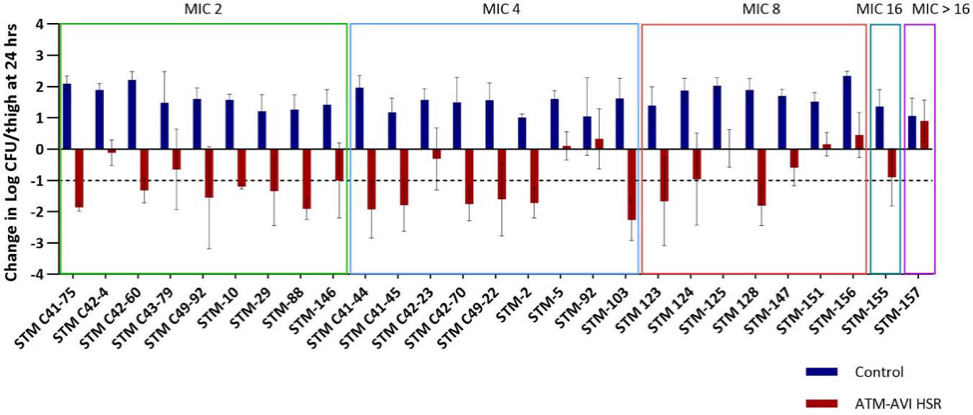
In vivo efficacy of ATM-AVI human simulated exposure of 1.5g-0.5 g q6h 3 h infusion in the neutropenic murine thigh model against 27 Stenotrophomonas maltophilia

**Methods:**

The neutropenic murine thigh infection model was employed to evaluate 27 STM with ATM-AVI MICs between 2/4 to > 16/4 mg/L; all isolates were resistant to ATM alone with MIC > 64 mg/L. Mice received cyclophosphamide on day -4 and day -1 before study bacteria inoculation and uranyl nitrate on day -3 to induce a predictable renal impairment. A Human Simulated Regimen (HSR) of ATM-AVI 1.5g-0.5 g q6h (3 h infusion) was developed to mimic the free plasma concentration profile from patients for both drugs. Six mice per isolate were sacrificed 2 h after inoculation to determine the baseline bacterial burden, and 12 mice (6 in control and 6 in ATM-AVI HSR group) per isolate were sacrificed 24 h after. Efficacy of the ATM-AVI HSR was defined as a mean decrease in colony forming units (CFU) of at least 1 log_10_ CFU/thigh at 24 h compared with baseline bacterial burden.

**Results:**

Mean baseline CFU for the 27 STM in the model was 6.22 ± 0.16 log_10_ CFU/thigh. STM grew on average to 7.81 ± 0.44 log_10_ CFU/thigh in control mice. The change of CFU/thigh data at 24 h for control (blue) compared with ATM-AVI treated (red) mice are displayed in Figure 1. Among STM isolates, 78% (7/9), 67% (6/9), 29% (2/7), and 0% (0/2) reached > 1 log_10_ CFU/thigh reduction at MICs of 2/4, 4/4, 8/4 and ≥ 16/4 mg/L, respectively.

**Conclusion:**

This study provides a translational assessment of *in vivo* efficacy for ATM-AVI against STM. At least 1 log_10_ CFU/thigh reduction was observed for the majority of STM isolates up to an ATM/AVI MIC of 4/4 mg/L. These data will be useful when determining susceptibility breakpoints for ATM/AVI against STM.

**Disclosures:**

David P. Nicolau, PharmD, CARB-X: Grant/Research Support|Innoviva: Advisor/Consultant|Innoviva: Grant/Research Support|Shionogi: Advisor/Consultant|Shionogi: Grant/Research Support Joseph L. Kuti, PharmD, Abbvie: Advisor/Consultant|Abbvie: Grant/Research Support|Abbvie: Honoraria

